# Construction of advanced producers of first- and second-generation ethanol in *Saccharomyces cerevisiae* and selected species of non-conventional yeasts (*Scheffersomyces stipitis, Ogataea polymorpha*)

**DOI:** 10.1007/s10295-019-02242-x

**Published:** 2019-10-21

**Authors:** Justyna Ruchala, Olena O. Kurylenko, Kostyantyn V. Dmytruk, Andriy A. Sibirny

**Affiliations:** 1 Department of Microbiology and Biotechnology, University of Rzeszow, Zelwerowicza 4, 35-601 Rzeszow, Poland; 2 Department of Molecular Genetics and Biotechnology, Institute of Cell Biology, NAS of Ukraine, Drahomanov Street, 14/16, Lviv, 79005 Ukraine

**Keywords:** Alcoholic fermentation, Bioethanol, Yeasts, Metabolic engineering

## Abstract

This review summarizes progress in the construction of efficient yeast ethanol producers from glucose/sucrose and lignocellulose. *Saccharomyces cerevisiae* is the major industrial producer of first-generation ethanol. The different approaches to increase ethanol yield and productivity from glucose in *S. cerevisiae* are described. Construction of the producers of second-generation ethanol is described for *S. cerevisiae*, one of the best natural xylose fermenters, *Scheffersomyces stipitis* and the most thermotolerant yeast known *Ogataea polymorpha*. Each of these organisms has some advantages and drawbacks. *S. cerevisiae* is the primary industrial ethanol producer and is the most ethanol tolerant natural yeast known and, however, cannot metabolize xylose. *S. stipitis* can effectively ferment both glucose and xylose and, however, has low ethanol tolerance and requires oxygen for growth. *O. polymorpha* grows and ferments at high temperatures and, however, produces very low amounts of ethanol from xylose. Review describes how the mentioned drawbacks could be overcome.

## Introduction

Global energy demands and environmental problems have stimulated efforts for biofuels’ production from renewable resources, which will be used to supplement gasoline from fossil fuel (oil) [109]. Due to the current political instability in oil-producing nations, and the concerns about global warming (attributed to harmful greenhouse gas emissions), there has been a call for greater reliance on domestic energy sources for the development of economically viable and environmentally friendly biofuels from cellulosic resources.

Fuel ethanol is a renewable liquid transportation fuel widely used in the United States and Brazil that is expected to become a dominant renewable biofuel in the transport sector within the next 20 years [42, 56, 123]. Most ethanol is currently made from maize (US) or sugar cane (Brazil), but future supplies could be derived from cellulosic and hemicellulosic sugars found in herbaceous biomass and agricultural residues. This can be documented by the worldwide exponential growth in ethanol production during the last decade, reaching 120 billion liters in 2018, 100 billion liters of this amount representing fuel ethanol (https://knect365.com/energy/article/c07f7fba-48fa-464f-9f21-12f913fc67f7/world-ethanol-production-to-expand-steadily-in-2019). Ethanol can be blended with gasoline or used as a neat alcohol in dedicated engines, taking advantage of the higher octane number and higher heat of vaporization. Furthermore, it is considered as an excellent fuel for advanced flexi-fuel hybrid vehicles.

Below, we describe different approaches towards construction of more robust and efficient producers of first- and second-generation ethanol in conventional (*Saccharomyces cerevisiae*) and non-conventional (*Scheffersomyces stipitis, Ogataea polymorpha*) yeasts.

### *Saccharomyces cerevisiae*

#### First-generation ethanol

Today, industrial-scale production of fuel ethanol uses primarily conventional feedstocks such as glucose (derived from starch) and sucrose (from sugarcane or sugarbeet), and is known as first-generation (1G) ethanol.

*S. cerevisiae* is the organism of choice for industrial ethanol production. The ethanol fermentation is the largest industrial biotechnological application of yeast. *S. cerevisiae* is characterized by several desirable industrial properties which include fast growth, efficient glucose anaerobic metabolism, high ethanol productivity, high yield, and high tolerance to several environmental stress factors, such as ethanol, low pH, and low oxygen. In addition, yeasts in general are insensitive to bacteriophage infection, which is particularly relevant in large industrial processes that use bacteria as the production microorganism. During alcoholic fermentation, *S. cerevisiae* produces ethanol with a yield close to the theoretical maximum (0.51 g ethanol per g of consumed glucose) [48]. At industrial scale, ethanol is produced with a yield that is higher than 90% of the theoretical maximum [28]. Taking into account the large worldwide ethanol production, an increase of ethanol yield of even 1% can provide additional estimated profits that approach hundreds of millions of dollars annually.

*S. cerevisiae* catabolizes glucose via the Embden–Meyerhof–Parnas (EMP) pathway yielding anaerobically 2 mol ATP per mole of consumed glucose. The efficiency of this pathway for anabolic processes is low with a maximal biomass yield of around 7% and an ethanol yield in the range of 90–93% from the glucose consumed [68]. In contrast to *S. cerevisiae*, the bacterium, *Zymomonas mobilis,* ferments glucose through the Entner–Doudoroff (ED) pathway. This pathway provides only 1 mol of ATP per mole of glucose, and consequently directs only 3% of glucose used to biomass production, while the remaining 97% is converted to ethanol at almost the possible theoretical value [171]. When ATP is used for growth, cell biomass is formed at the expense of glucose that is not converted to ethanol. In other words, cell biomass is a byproduct that is produced during alcoholic fermentation. Therefore, the lowering of ATP yield during alcoholic fermentation increases ethanol yield with reduced substrate conversion to cell mass. To achieve this goal, several strategies can be applied such as: (i) substituting the EMP pathway in yeast by the ED pathway; (ii) activation of energy-consuming plasma membrane sugars symporters; (iii) generation of futile cycles; and (iv) elevating activity of ATP-degrading enzymes [27]. Another possibility is to decrease production of glycerol, which is another major by-product of ethanol production in yeast [48]. This is somewhat more difficult, because glycerol accumulation results from a redox imbalance generated during cell growth.

Notwithstanding the former patent application of Lancashire et al. [104] which describes the functional integration of the ED pathway to bypass glycolysis in *S. cerevisiae* with positive impact on ethanol yield, more recent work demonstrates the inability to express in this yeast one of the two unique enzymes to the ED pathway, namely, 6-phosphogluconate dehydratase (PGDH) [16]. Several attempts to improve the availability of iron–sulfur clusters in the yeast cells and to attract the iron–sulfur cluster assembly machinery to PGDH protein did not result in improved enzyme activities [16].

The impact of the disaccharide–proton symport on biomass and ethanol yields was studied in *S. cerevisiae* by comparing anaerobic growth on maltose, which is transported by a maltose–proton symporter and intracellularly hydrolyzed by maltase, to that of glucose [196]. Biomass and ethanol yields in anaerobic maltose-limited cultures were 25% lower and 8% higher, respectively, than that of glucose-limited cultures. Relocation of sucrose hydrolysis from the extracellular space to the cytosol with additional evolutionary engineering resulted in a strain that had elevated sucrose uptake kinetics with a 30% decrease in the biomass yield and an 11% increase in the ethanol yield relative to the reference strain. The evolved strain showed an increased transcript level for *AGT1* gene-encoded sucrose–proton symporter [14]. The improved kinetics of sucrose transport with concomitant ATP reduction could be achieved by targeted overexpression of *AGT1* or the heterologous genes encoding sucrose–proton symporters from yeasts that naturally hydrolyze sucrose intracellularly [81]. Replacement of *S. cerevisiae* facilitated diffusion systems by heterologous proton symporters for the other industrially relevant sugars (e.g., glucose) should drop the biomass accumulation and elevate the ethanol yield [27].

Another approach which could lead to increasing the ethanol yield is based on the use of a futile cycle, i.e., a set of at least two biochemical reactions that run simultaneously in opposite directions and one of which splits ATP, thus resulting in energy dissipation. A classical example of the futile cycle is combined expression of phosphofructokinase and fructose-1,6-bisphosphatase. Phosphofructokinase is a glycolytic enzyme that phosphorylates fructose 6-phosphate to fructose-1,6-bisphosphate with ATP expediture. Fructose-1,6-biphosphatase (FBPase) is one of the specific gluconeogenesis enzymes. It hydrolyzes fructose-1,6-bisphosphate to fructose-6-phosphate with no ATP production. The simultaneous action of both enzymes—phosphofructokinase and fructose-1,6-biphosphatase—leads to a futile cycle resulted in ATP dissipation. Phosphofructokinase is primarily regulated at the level of activity and the differences in the amount of the enzyme in glycolytic and gluconeogenic conditions do not exceed twofold [8, 23]. In contrast, the FBPase activity is tightly regulated by catabolic repression, inactivation through ubiquitination, inhibition by AMP and fructose-2,6-biphosphate [126, 127]. Therefore, intracellular FBPase activity level is maintained at basal level in cells grown in media containing fermentable carbon sources. To overcome the tight regulation of the yeast FBPase, the bacterial FBPase from *Escherichia coli* that is insensitive to fructose-2,6-biphosphate inhibition was constitutively expressed in yeast [127, 164]. The resulting recombinant strain exhibited a threefold increase in FBPase activity, a 30% reduced intracellular ATP level and up to 9% increase of ethanol production relative to that of parental strain [164].

An alternate futile cycle was generated via the simultaneous activation of two enzymes, pyruvate carboxylase and the gluconeogenic enzyme, phosphoenolpyruvate carboxykinase [164]. Pyruvate carboxylase catalyzes the conversion of pyruvate into oxaloacetate in an ATP-dependent reaction, and phosphoenolpyruvate carboxykinase uses ATP energy to convert oxaloacetate into phosphoenolpyruvate. ATP is synthesized when phosphoenolpyruvate is converted into pyruvate-by-pyruvate kinase. Therefore, the resulting total loss in ATP is one molecule for each one turn of the cycle. The specific activity of pyruvate carboxylase was increased 3–5-fold via substitution of target gene promoter with a strong constitutive one. The activity of phosphoenolpyruvate carboxykinase is regulated at the post-translational level [198]. To avoid such regulation, a heterologous gene coding for the corresponding enzyme from *E. coli* was overexpressed. As a result, the specific activity of phosphoenolpyruvate carboxykinase was elevated 6–7 fold. Ethanol production by the constructed recombinant strains revealed a twofold increase over the parental strain by the end of the first day of fermentation [164]. Potential futile cycle based on the interconversion between glucose and trehalose, which theoretically could lead to ATP dissipation in cells of *S. cerevisiae* was generated by the activation of trehalose-6-phosphate synthase and the neutral trehalase responsible for the synthesis and degradation of trehalose [164]. Despite the increase in the activities of both enzymes, biomass accumulation remained unchanged.

Lowering the intracellular ATP content in yeast cell with concomitant increase in ethanol yield can also be achieved by the activation of some of the cytosolic ATPases. A decrease in cellular ATP pool and activation of alcoholic fermentation was achieved by the overexpression of the soluble part (F_1_) of H^+^-ATPase or a portion of F_1_ exhibiting ATPase from different origins in *S. cerevisiae* [75]. Similar results were obtained after the overexpression of *PHO5* coding for acid phosphatase which is a non-specific enzyme that also hydrolyzes ATP [158]. Overexpression of the vacuolar alkaline phosphatase Pho8 led to increase in yield and ethanol production from glucose in both a laboratory and an industrial strains of *S. cerevisiae* (Table 1), whereas the expression of the truncated cytosol localized form is detrimental to cell growth [163]. The galactose induced expression of ATP hydrolysis region of the ribosome-associated molecular chaperon encoded by gene *SSB1* of *S. cerevisiae* and the ATP-diphosphohydrolases also known as apyrases from *E. coli,* resulted in an increase in ethanol yield of 39 and 29%, respectively, during fermentation of the corresponding recombinant *S. cerevisiae* strains in galactose containing media [164]. Thus, creation of functional futile cycles or overexpression of ATPases could be the promising approaches for enhancing the ethanol yield in yeast.

**Table 1:** Growth rate, ATP level, alkaline phosphatase activity ethanol productivity and yield of *S. cerevisiae* transformants and control strains

Strain	Specific growth rate, g l^−1^h^−1^	ATP, μmoles of ATP mg^−1^ dry cell weight	Alkaline phosphatase activity, nmoles of product mg^−1^ of prot min^−1^	Ethanol productivity		Ethanol yield g g^−1^ of consumed glucose
				g l^−1^h^−1^	g l^−1^/g^−1^ biomass/h^−1^	
BY4742	0.031 ± 0.002	7.95 ± 0.10	85.2 ± 3.3	0.79 ± 0.014	0.3 ± 0.006	0.379 ± 0.007
BY4742/Pho8vac	0.032 ± 0.001	7.83 ± 0.06	1948.3 ± 175.3	0.92 ± 0.019	0.34 ± 0.007	0.442 ± 0.009
BY4742/Pho8cyt	0.025 ± 0.001	7.56 ± 0.08	1832.4 ± 137.4	0.42 ± 0.004	0.18 ± 0.002	0.202 ± 0.002

Cells of BY4742 strain and its PHO8-expressing derivatives were grown in 100 ml of YPD medium in Erlenmeyer flasks (bottle size—300 ml) overnight and then used to inoculate a 20 ml of YNB medium with 100 g/l glucose in 50 ml Erlenmeyer flasks. An initial biomass concentration of 1.2 g (dry weight)/L was used for fermentation. Fermentation was carried out at a temperature of 30 °C with limited aeration using a gyratory shaker at a setting of 120 revolutions/min

(±) absolute error

Glycerol is the second primary byproduct after cell biomass during the ethanol production. Glycerol is formed at the expense of sugar that is not converted to ethanol [48]. In yeast, the reduction of glycerol formation can most probably result in an increase in ethanol yield. Therefore, significant research efforts have been directed towards reducing glycerol formation during fermentation. This can be accomplished by deleting one or both genes *GPD1* and *GPD2*, coding for glycerol-3-phosphate dehydrogenase [7]. Deletion of both genes affected anaerobic growth. Deletion of *GPD2* resulted in an increase in ethanol yield with concomitant decrease in glycerol production; however, this deletion also reduced growth and ethanol productivity [180]. Glycerol formation results from the regeneration of NAD from excess NADH produced during glycolysis under anaerobic conditions. To decrease cytosolic NADH formation, the gene *GDH1* encoding NADPH-dependent glutamate dehydrogenase was deleted, while *GLN1* and *GLT1* coding for glutamine synthetase and NAD-dependent glutamate synthase were overexpressed. During ammonium assimilation that is linked with NADH and ATP consumption, a recombinant strain decreased the glycerol yield by 38% while increasing the ethanol yield by 10% [134]. Another approach for reducing the intracellular pool of NADH and ATP production consisted of replacing the glyceraldehyde-3-phosphate dehydrogenase gene by that of the non-phosphorylating heterologous NADP-dependent analog from *Bacillus cereus*, or *Streptococcus mutans* [54, 208]. The combination of this approach with the overexpression of NAD-dependent fumarate reductase or acetaldehyde dehydrogenase increased ethanol yield to 95% of the theoretical maximum [208].

Other work demonstrated that the expression of the NAD-dependent acetaldehyde dehydrogenase from *E. coli* in an *S. cerevisiae gpd1Δ gpd2Δ* strain supports the growth in medium with glucose supplemented with acetate under anaerobic conditions [51]. The constructed strains could be used for alcoholic fermentation of acetate containing substrates, as they are able to convert acetate into ethanol. The main drawback of these *S. serevisiae* strains is that the reduced glycerol production resulted also in reduced osmotolerance and overall viability [63]. This finding imposes certain restrictions on the application of these strains in industrial fermentation processes that normally run at high substrate concentration. Other significant efforts have focused on improving the stress resistance of yeast strains with reduced glycerol production. Osmotolerance, thermotolerance, and tolerance to high concentrations of ethanol of several target strains were elevated by applying methods of metabolic engineering and genomic shuffling [64, 189]. The combination of the deletion of the *GPD1* gene with the overexpression of the *nadF* gene from *B. cereus* that encodes for an NADP-dependent glyceraldehyde-3-phosphate dehydrogenase with the derepression of homologous genes of trehalose synthesis *TPS1* and *TPS2* (encoding trehalose-6-phosphate synthase and trehalose-6-phosphate phosphatase), resulted in increased ethanol production, and reduced glycerol formation, but did not exhibit a negative influence on strains viability during alcoholic fermentation [54].

Trehalose protects cells from stress and its intracellular concentration correlates with resistance to high temperatures and high concentrations of ethanol in the medium [176]. Overexpression of the *TPS1* gene in *S. serevisiae* resulted in increased thermotolerance, thereby allowing for the possible reduction in energy costs for cooling of fermentation vessels as well as for savings in energy used for heating due to reduced temperature difference between fermentation and distillation processes [6]. Moreover, high-temperature fermentation has advantages during the process of simultaneous saccharification and fermentation, as high temperature (around 50 °C) is beneficial to hydrolytic enzymes involved in saccharification [165].

The development of improved ethanol-producing strains can be achieved by applying traditional selection and adaptive evolution as a useful alternative to metabolic engineering approaches. Mutants resistant to toxic concentrations of oxythiamine, trehalose, 3-bromopyruvate, glyoxylic acid, and glucosamine have been isolated. Some of these are characterized by 5–8% increase in ethanol yield when compared to the parental industrial ethanol-producing strain (Table 2) [29]. By applying adaptive evolution, useful yeast strains with enhanced maltose utilization and osmotolerance [62] increased ethanol tolerance [174] and yeast with improved ethanol production rate and decreased formation of acetate were selected [21].

**Table 2:** Growth rate, ethanol production, productivity, specific productivity and yield of *S. cerevisiae* mutants resistant to oxythiamine, trehalose, bromopyruvate, glyoxylic acid, glucosamine and initial industrial strain AS400 during alcoholic fermentation of on YNB medium supplemented with 20% glucose and corn steep liquor (CSL) medium with hydrolyzed meal indicated in brackets

Strain	Selective agent	Specific growth rate, g l^−1^h^−1^	Ethanol, g l^−1^	Ethanol productivity, g l^−1^h^−1^	Specific ethanol productivity, g g^−1^ biomass h^−1^	Ethanol yield, g g^−1^ of consumed glucose
AS400	–	0.133 ± 0.003	80.3 ± 1.5 (84.6 ± 1.0)	4.46 ± 0.08 (3.53 ± 0.03)	1.86 ± 0.03	0.402 ± 0.007 (0.423 ± 0.005)
AS400-567	Oxythiamine	0.133 ± 0.004	85.2 ± 1.6 (90.5 ± 0.9)	4.73 ± 0.09 (3.77 ± 0.04)	1.97 ± 0.04	0.426 ± 0.008 (0.453 ± 0.004)
AS400-543	Trehalose	0.083 ± 0.002	84.3 ± 1.6 (88.8 ± 0.9)	4.68 ± 0.09 (3.70 ± 0.03)	2.75 ± 0.06	0.422 ± 0.008 (0.444 ± 0.005)
AS400-617	Bromopyruvate	0.133 ± 0.003	84.1 ± 1.5 (89.7 ± 1.0)	4.67 ± 0.09 (3.74 ± 0.03)	1.95 ± 0.03	0.421 ± 0.007 (0.449 ± 0.005)
AS400-510	Glyoxylic acid	0.133 ± 0.004	85.1 ± 1.6 (91.4 ± 0.9)	4.73 ± 0.09 (3.81 ± 0.03)	1.97 ± 0.04	0.426 ± 0.007 (0.457 ± 0.005)
AS400-128	Glucosamine	0.117 ± 0.003	84.6 ± 1.7 (89.6 ± 0.9)	4.70 ± 0.09 (3.73 ± 0.04)	2.24 ± 0.04	0.423 ± 0.008 (0.448 ± 0.005)
AS400-510-42	Glyoxylic acid, glucosamine	0.133 ± 0.004	86.7 ± 1.7 (93.0 ± 0.9)	4.82 ± 0.09 (3.88 ± 0.03)	2.01 ± 0.04	0.434 ± 0.008 (0.465 ± 0.004)
AS400-510-42-214	Glyoxylic acid, glucosamine, bromopyruvate	0.117 ± 0.003	88.4 ± 1.5 (94.8 ± 1.0)	4.91 ± 0.08 (3.95 ± 0.04)	2.34 ± 0.04	0.442 ± 0.008 (0.474 ± 0.005)

For alcoholic fermentation, cells of AS400 strain and its derivatives were grown overnight in 100 ml of YPD medium in 300 ml Erlenmeyer flasks and then used to inoculate 20 ml aliquots of mineral YNB medium supplemented with 200 g/l glucose or CSL medium supplemented with hydrolyzed meal in 50 ml Erlenmeyer flasks. An initial biomass concentration of 8 g (dry weight)/l was used for fermentation in YNB medium. For CSL medium, an initial biomass concentration of 10 g (dry weight)/l was used. Fermentation was carried out at a temperature of 34 °C with limited aeration using a gyratory shaker at a setting of 120 rpm. Samples were taken every 3 h for YNB medium or 12 h for CSL medium

(±) absolute error

#### Second-generation ethanol

In contrast to 1G, the 2G ethanol is ethanol produced from non-food feedstocks such as dried plant biomass (lignocellulose). The utilization of lignocellulosic biomass for 2G ethanol production would be preferable over sugar and starch-based 1G ethanol production because of significantly lower competition with food and animal feed production and minimal changes to land use [131, 161]. 2G ethanol can utilize a range of different types of lignocellulosic substrates. Currently, a limited amount of 2G ethanol is produced at several pilot and demonstration plants around the world; however, due to higher cost of the large-scale ethanol production from lignocellulosics, it is not yet commercially feasible [40, 109, 135].

The technologies to produce 2G ethanol do exist; however, many improvements are needed. The production of 2G ethanol from lignocellulosics will require the development of robust microbial strains that can grow and produce ethanol from at least glucose and xylose, which are the major fermentable sugars produced from the hydrolysis of lignocellulosic biomass. Other sugars that are produced during hydrolysis include hexoses fructose, mannose, galactose, and the pentose sugar l-arabinose.

The yeast *S. cerevisiae* efficiently ferments hexoses glucose, fructose, and mannose but to a lesser extent galactose. In this yeast, galactose transporters are subjected to catabolic repression by glucose, thereby limiting co-fermentation of these sugars. In contrast, mannose enters yeast cells using the glucose transport system allowing glucose/mannose co-fermentation to ethanol [118]. The combined deletion of the genes *GAL6*, *GAL80,* and *MIG1* involved in negative regulation of galactose catabolism in a laboratory *S. cerevisiae* strain resulted in a partial co-consumption of glucose and galactose in aerobic batch cultures [136]. Normally, galactose metabolism in *S. cerevisiae* requires respiration. However, a *cox9*Δ *gal80*Δ double mutant has been isolated which effectively fermented galactose anaerobically [150]. A natural strain of *S. cerevisiae* (NRRL Y-1528), which catabolizes galactose more effectively than glucose or mannose and is capable of simultaneously fermenting all hexoses present in hydrolyzed biomass was described. This strain has a proposed deficiency in carbon catabolic repression, which avoids glucose repression of galactose utilization [84].

**Fig. 1:**
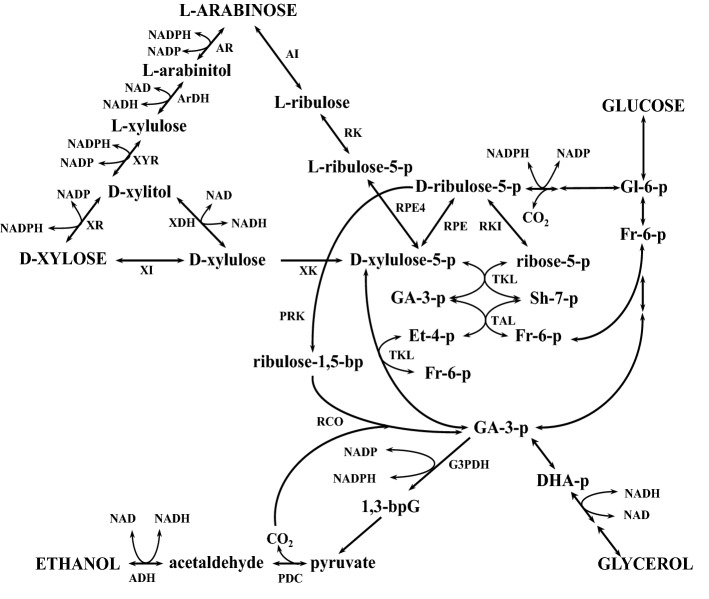
Pathways of xylose, l-arabinose and glucose fermentation to ethanol in yeasts. Gl-6-p is glucose 6-phosphate; Fr-6-p is fructose 6-phosphate; GA-3-p is glyceraldehyde 3-phosphate; SH-7-p is sedoheptulose 7-phosphate; ET-4-p is erythrose 4-phosphate; 1,3-bpG is 1,3-bisphospho-glycerate; DHA-p is dihydroxyacetone phosphate; XR is xylose reductase; XDH is xylitol dehydrogenase; XI is xylose isomerase; XK is xylulokinase; AI is arabinose isomerases; RK is ribulokinase; RPE4 is ribulose-5-phosphate-4-epimerase; AR is aldose reductase; ArDH is arabinitol dehydrogenase; XYR is xylulose reductase; RKI is ribose-5-phosphate ketol-isomerase; RPE is ribulose-5-phosphate-3-epimerase; TKL is transketolase; TAL is translaldolase; PRK is phosphoribulokinase; RCO is ribulose-1,5-bisphosphate carboxylase/oxygenase; G3PDH is glyceraldehyde 3-phosphate dehydrogenase; PDC is pyruvate decarboxylase; ADH is alcohol dehydrogenase

Wild-type strains of *S. cerevisiae* are unable to catabolize and ferment pentoses (d-xylose or l-arabinose, referred to further as xylose and arabinose, respectively) which are major constituents of hydrolysates of plant biomass. This is consequence of the absence of the enzymes that catalyze the initial stages of pentose catabolism. Since the xylose content of plant biomass hydrolysates is significantly higher than that of arabinose, most work has been directed to the construction of *S. cerevisiae* strains capable of xylose catabolism and fermentation. These efforts have been focused on the functional expression of the heterologous genes for xylose catabolism of prokaryotic and eukaryotic origin. Some efforts have been focused on the expression of the genes encoding xylose isomerase (XI) from different microorganisms. This enzyme does not require cofactors and catalyzes the isomerisation of xylose in xylulose (Fig. 1). Several successful attempts have been made to express XI from the bacteria *Thermus thermophilus* [188], *Clostridium phytofermentans* [20], *Bacteroides stercoris* [55], or from the anaerobic fungus *Piromyces* sp. E2 [82] or from *Orpinomyces* sp. [117]. Expression of the codon-optimized XI of *C. phytofermentans* in *S. cerevisiae* resulted in a 46% increase in specific growth rate on xylose as compared to the strain expressing a non-optimized version of the gene [20]. XI from the bacterium *Propionibacterium acidipropionici* became functionally active in *S. cerevisiae* when co-expressed with GroEL–GroES chaperonin complex from *E. coli* [178]. More details on functional expression if XI in *S. cerevisiae* described in a recent reviews [72, 103].

Many studies have been focused on the expression of heterologous yeast genes encoding xylose reductase (XR) and xylitol dehydrogenase (XDH), which catalyze the conversion of xylose into xylitol and subsequently to xylulose (Fig. 1). XR is encoded by *XYL1* which can use either NADH or NADPH as cofactors but which generally shows higher affinity to NADPH. XDH is an NAD- and Zn-dependent secondary alcohol dehydrogenase that is encoded by *XYL2*. The co-expression of both genes resulted in an imbalance of cofactors with a deficiency in NAD and an excess of NADP. It is assumed that this imbalance causes the low efficiency of xylose alcoholic fermentation, primarily due to the xylitol accumulation [77, 94]. A genome-wide scale model of *S. cerevisiae* was used to predict the maximal achievable growth rate for cofactor balanced xylose utilization pathway. By applying the use of dynamic modeling with experimental results, a balanced cofactor system XR/XDH showed a 24.7% increase in ethanol batch production and simultaneously reduced the predicted substrate utilization time by up to 70% [47]. The combination of computational design [85] and site-specific mutagenesis of domains responsible for binding these cofactors [192], resulted in a modification of the cofactor specificity of XR and XDH and for balanced action. The co-expression of the mutant forms of XR from *S. stipitis* (K270 M, K270R, K270R/N272D, N272D/P275Q, R276H) with elevated affinity to NADH along with a native NAD-dependent XDH increased productivity and ethanol yield with concomitant reduction of xylitol yield in recombinant strains of *S. cerevisiae* [15, 108]. A decrease in xylitol and an increase in ethanol yields were also reported as a result of co-expression of the native XR with the modified XDH (D207A/I208R/F209S/N211R) which exhibits affinity to NADP. Xylose consumption was also 32% faster when compared to the strain expressing wild-type alleles of XR and XDH [120]. The ratio between XR and XDH activities is essential to improve xylose alcoholic fermentation. The low level of *XYL2* gene expression is the main reason for the accumulation of xylitol in strains expressing *XYL1*, *XYL2,* and *XYL3* [90].

Comparison of the industrial *S. cerevisiae* with XI and/or XR/XDH pathways regarding xylose fermentation efficiency showed that XI-expressing strains are advantageous regarding XR/XDH expressing strains; however, the most efficient xylose fermentation was found in the recombinant strain which simultaneously expresses both XI and XR/XDH pathways [25]. Co-expression of both pathways significantly improved ethanol production from non-detoxified hemicellulosic hydrolysates.

Xylulokinase (XK) catalyzes the ATP-dependent phosphorylation of xylulose and is an important enzyme of xylose catabolism (Fig. 1). XK catalyzes the limiting step in xylose alcoholic fermentation as *S. serevisiae* strains expressing XR/XDH or XI have a significant reduction in xylitol accumulation when XK was overexpressed [139]. The activation of XK was accomplished either by the derepression of the homologous *S. cerevisiae* gene *XKS1* or by heterologous expression of the *XYL3* gene from *S. stipitis* [79]. However, high levels of XK activity have a negative impact on the growth of recombinant strains on xylose, which is probably due to a rapid depletion of ATP caused by xylulose phosphorylation [35].

To further increase ethanol production, xylose-fermenting strains of *S. serevisiae* expressing the genes for xylose catabolism were subjected to other metabolic modifications. In particular, the heterologous expression of genes for the sugar transporters Sut1 and Sut2 from *S. stipitis* was carried out [35, 61]. A system for the selection of glucose transporters with high affinity for xylose has been also developed. As a result, a modified version of the Gal2 *S. cerevisiae* transporter that is able to transport only xylose was constructed [41]. It was also shown that endogenous *S. cerevisiae* hexose transporters (Hxt) can be engineered into specific xylose transporters [41, 132]. However, xylose transporters synthesized in these strains remain subjected to protein degradation. For example, some Hxt proteins with high xylose transport capacity that are rapidly degraded in the absence of glucose or when glucose is exhausted from the medium [133]. The N-terminal lysine residues of the Hxt proteins were predicted to be the target of ubiquitination. The substitution of N-terminal lysine residues in the hexose transporters Hxt1 and Hxt36, which are subjected to catabolite degradation, resulted in improved retention of these transporters at the cytoplasmic membrane in the absence of glucose and improved xylose fermentation upon the depletion of glucose when cells were grown in xylose alone [133]. An interesting approach proposed to create an artificial hybrid protein consisting of a sugar transporter and xylose isomerase. Such hybrid protein was successfully expressed in *S. cerevisiae* and the resulting strain showed acceleration of xylose consumption and substantially diminished production of xylitol as an undesired side product, with a concomitant increase in the production of ethanol [179].

To achieve efficient glucose/xylose co-fermentation, a modified xylose-specific, glucose-insensitive transporter Mgt05196 (N360F) from *Pichia (Meyerozyma) guilliermondii,* was expressed in the background of an xylose-fermenting *S. cerevisiae* strain overproducing xylose isomerase XI, xylulokinase Xks1 and the enzymes of non-oxidative pentose phosphate pathway (PPP), and in which the aldose reductase Gre3p and the alkaline phosphatase Pho13 had been inactivated. These rationally designed genetic modifications, combined with alternating adaptive evolution in xylose and lignocellulosic hydrolysates, resulted in a final strain, with excellent xylose fermentation that had an enhanced resistance to inhibitors [110]. Glucose/xylose co-fermentation was activated after *HXK2* deletion and introduction of a *GAL83* G673T allele which provided 2.5-fold higher xylose and glucose co-consumption ratio than its xylose-fermenting parental strain [138].

The co-expression of genes of non-oxidative part of PPP (*RKI1, RPE1, TKL1*, and *TAL1*, encoding ribose-5-phosphate isomerase, ribulose-5-phosphate epimerase, transketolase, and transaldolase, respectively) (Fig. 1) on the background of a *S. cerevisiae* strain expressing XI and XK, resulted in improved growth on xylose [43, 83]. In a recombinant strain expressing XI, xylose consumption and fermentation were also enhanced after homologous or heterologous (from thermotolerant yeast *Kluyveromyces marxianus*) overexpression of *RKI1, TAL1* and *TKL1* genes [93]. However, expression of genes of the non-oxidative part of PPP in an *S. cerevisiae* strain expressing XR, XDH, and XK did not improve its growth on a medium supplemented with xylose [17].

NADPH synthesis mainly occurs in the oxidative part of PPP. A series of studies were conducted to reduce the activity of the enzymes participating in the oxidative branch of PPP. This approach was hypothesized to facilitate the reaction catalyzed by XR using NADH instead of NADPH, with a reduced production of CO_2_. The deletion of genes *ZWF1* (encoding glucose-6-phosphate dehydrogenase) and/or *GND1* (decarboxylating 6-phosphogluconate dehydrogenase) with the simultaneous expression of the genes *XYL1* and *XYL2,* increased ethanol yield, but also significantly decrease xylose consumption rate during xylose fermentation [76]. Another successful approach describes the combination of fungal NADP-dependent glyceraldehyde-3-phosphate dehydrogenase overexpression in part with the deletion of the *ZWF1* gene, resulting in an increase in ethanol yield and productivity during xylose fermentation [184]. To reduce xylitol production and increase ethanol yield during fermentation of glucose/xylose mixture, a modification of the redox balance of recombinant strains of *S. cerevisiae* was carried out by the deletion of gene *GDH1* (coding for NADPH-dependent glutamate dehydrogenase) and the overexpression of *GDH2* gene (NADH-dependent glutamate dehydrogenase) responsible for ammonium assimilation [156]. Xylitol accumulation was reduced by overexpressing the *noxE* gene from *L. lactis*, which codes for the water forming NADH-oxidase [206]. Modification of the acetate biosynthetic pathway involving deletion of the *ALD6* gene encoding NADP-dependent aldehyde dehydrogenase also increased the efficiency of xylose alcohol fermentation [108].

To increase the production of ethanol during xylose fermentation, recombinant strains of *S. cerevisiae* were subjected to adaptive evolution and genome shuffling [80, 90, 152]. Sequencing of the genomes of these strains after long-term culturing in xylose containing media revealed new potential target genes for metabolic engineering approach, e.g., *XKS1, SOL3* (6-phosphogluconolactonase), *GND1* (6-phosphogluconate dehydrogenase), *TAL1, TKL1, YCR020C, YBR083* *W,* and *YPR199C* [187]. The genes for *TAL1* and *PHO13* (non-specific alkaline phosphatase) were identified by transposon mutagenesis as new targets. The truncation or deletion of *PHO13* particularly increased production of ethanol during xylose fermentation [13, 90, 91, 103, 110, 129].

Carbon dioxide is a major by-product of carbohydrate alcoholic fermentation. An attractive target for manipulation is conserving carbon that is released from the decarboxylation step of pyruvate into acetaldehyde in the last stage of the ethanol-producing pathway. This goal was achieved by the introduction a synthetic reductive PPP into a xylose-fermenting *S. cerevisiae* strain, which resulted in simultaneous lignocellulosic ethanol production and carbon dioxide recycling. The heterologous enzymes phosphoribulokinase from *Spinacia oleracea* and ribulose-1,5-bisphosphate carboxylase/oxygenase from *Rhodospirillum rubrum,* were introduced into the *S. cerevisiae* strain harboring XR/XDH pathway, up-regulated PPP and knocked out *ALD6* and *PHO13* genes [90]. The phosphoribulokinase enzyme catalyzes ribulose-5-phosphate conversion to ribulose-1,5-bisphosphate, and the ribulose-1,5-bisphosphate carboxylase/oxygenase converts one ribulose-1,5-bisphosphate and CO_2_ to two molecules of glycerate-3-phosphate (Fig. 1). The constructed strain with the synthetic reductive PPP revealed a higher yield of ethanol with lower yields of xylitol and glycerol than that of the parental strain. Moreover, a reduced release of CO_2_ by the engineered strain was observed during xylose fermentation, suggesting that the carbon dioxide generated by pyruvate decarboxylase was partially reassimilated through the synthetic reductive PPP [200]. The strategy of carbon dioxide recycling from the ethanol fermentation pathway in yeast has a great potential in alleviating greenhouse gas emissions during the production of 2G ethanol.

Alcoholic production from l-arabinose appears to be very important, as this pentose is present in hemicellulose and to even higher proportion in pectin [203]. Two different l-arabinose catabolizing pathways were identified in bacteria [199] and fungi [153, 154]. In the bacterial pathway, arabinose is isomerized to ribulose by arabinose isomerase (*araA*), and then, ribulose is phosphorylated to ribulose-5-phosphate with ribulokinase (*araB*) which in turn is converted to xylulose-5-phosphate by ribulose-5-phosphate-4-epimerase (*araD*) [199]. Arabinose fermentation was observed when *araA, araB,* and *araD* from *Lactobacillus plantarum* were co-expressed in *S. cerevisiae* [199, 203]. In fungi, l-arabinose is reduced to l-arabitol by aldose reductase and then is converted to l-xylulose with arabinitol dehydrogenase. l-xylulose is reduced to xylitol by xylulose reductase, which in turn is oxidized to xylulose by xylitol dehydrogenase (Fig. 1). The expression of the fungal arabinose utilization pathway (aldose reductase Gre3 from *S. cerevisiae*, arabinitol dehydrogenase, and xylulose reductase from *Trichoderma reesi*, XDH from *S. stipitis* and XK from *S. cerevisiae*) in *S. cerevisiae* resulted in low ethanol with substantial arabinitol production, which is apparently due to the imbalance caused by the cofactor mismatch among used reductases/dehydrogenases [18, 153, 154, 203]. Based on published results, the expression of genes of the bacterial redox-independent pathway for l-arabinose utilization in *S. cerevisiae* is a more promising approach [205].

Fermentation of biomass-derived hydrolysates is accompanied by the inhibition of ethanol production due to the presence of toxic compounds produced mainly during lignocellulose pretreatment, e.g., furfural, hydroxymethylfurfural (HMF), weak acids, and phenols [137]. To overcome this limitation, strains of *S. cerevisiae* that are able to produce ethanol in the presence of these inhibitors have been selected through directed evolution and adaptation [60, 140]. Other alternative molecular approaches were also applied. Furfural and HMF were converted to less toxic furfuryl alcohol and furan dimethanol, respectively, by the overexpression of the endogenous *S. cerevisiae* oxidoreductases such as alcohol dehydrogenases (*ADH1, ADH6,* and *ADH7*) [3, 113, 146] and aldo–keto reductases (*GRE2*) [124]. It was shown [49] that tolerance to furfural-induced stress is associated with genes of the pentose phosphate pathway *ZWF1*, *GND1*, *RPE1,* and *TKL1*. The overexpression of the gene *PAD1* encoding phenylacrylic-acid decarboxylase in *S. cerevisiae* resulted in improved growth rate and ethanol productivity in dilute-acid hydrolysates [106]. An original approach to improve tolerance to fermentation inhibitors and ethanol was proposed by modulation of the polyamine (spermidine) content in *S. cerevisiae* [89]. Intracellular spermidine contents were increased by a double gene deletion *OAZ1* (ornithine decarboxylase antizyme) and *TPO1* (polyamine transport protein) genes and the overexpression of *SPE3* (spermidine synthase) [89].

In summary, 1G ethanol production is a profitable technology that can be further improved by increasing ethanol yield and productivity by applying molecular and classical techniques to industrial ethanol-producing strains of *S. cerevisiae*. Despite significant efforts, existing technologies of 2G ethanol production are still non-profitable. Although recombinant *S. cerevisiae* strains capable of hexoses/pentoses co-fermentation were developed, none of the engineered strains are able to ferment glucose/xylose mixture as fast as the rate of glucose fermentation by the parental strain [205]. Another important limitation is the sensitivity of ethanol-producing strains to inhibitors in lignocellulosic hydrolysates. Further studies are needed to develop recombinant *S. cerevisiae* strains that are capable of rapid fermentation of mixed sugars with improved resistance to fermentation inhibitors.

### *Scheffersomyces (Pichia) stipitis*

*Scheffersomyces stipitis* (formerly known as *Pichia stipitis*) belongs to a group of yeasts that naturally ferment xylose while accumulating low amounts of by-products such as xylitol [97]. Other known representatives of the group of native xylose-fermenting yeastsare, *Candida shehatae* (teleomorph form is known as *Scheffersomyces shehatae*)*, Pachysolen tannophilus, Spathaspora passalidarum,* and *Ogataea polymorpha* [38, 128, 160, 162]. As a rule, most natural xylose-fermenting yeasts inhabit the guts of passalid beetles that degrade white-rotted hardwood [125, 130] with the exception of *O. polymorpha* strains that have been isolated from other natural habitats [46]. *S. stipitis* is closely related to other yeast endosymbionts of passalid beetles [175]. This yeast has one of the highest native capacities for xylose fermentation among yeast species described so far [39]. In addition to utilize xylose, *S. stipitis* has the capability to use all of the other major sugars found in wood. It also transforms low-molecular weight lignin moieties, reduces acyclic enones to the corresponding alcohols, and forms various esters and aroma components. It has been recently engineered to produce lactic acid or xylitol in high yield [24, 67, 87, 107, 177]. The published ethanol yield from fermented lignocellulosic sugars by adapted *S. stipitis* strain approached 80% of theoretical yield [130]. The availability of genetic tools and capability for fermentation of hydrolysates have made *S. stipitis* an attractive microorganism for bioconversion of lignocellulose to fuels and chemicals. The major drawbacks of *S. stipitis* are the low fermentation rates, low ethanol tolerance, and the inability to grow anaerobically [39, 50, 167].

*S. stipitis* is a predominantly haploid, homothallic, hemiascomycetous yeast that forms buds along with pseudomycelia during vegetative growth and two hat-shaped ascospores from each ascus [96]. Genetic manipulation of *S. stipitis* is much more complicated relative to those of *S. cerevisiae,* because *S. stipitis* is resistant to most common antibiotics and the number of strains with convenient auxotrophic markers is limited. This yeast uses an alternative codon system that decodes CUG for serine instead of leucine as common in the classical genetic code [155]. Thus, the expression of foreign proteins, including those used as drug resistance markers, requires codon modification. Unfortunately, random (non-homologous) integration prevails in *S. stipitis* which makes targeted deletions much more difficult to obtain [74]. Nevertheless, many efficient genetic methods have been developed for *S. stipitis* that include methods of sexual mating and sporulation [121]. Auxotrophic strains have been created and methods for high-efficiency transformation have been developed for the auxotrophic mutants *ura3, leu2, trp5,* and *his3* of *S. stipitis* [115, 148, 201]. Genetic tools based on a *loxP/Cre* recombination system and the dominant marker for zeocine resistance has been developed for *S. stipitis* [105]. Deletion of the *KU80* gene that is responsible for non-homologous end joining significantly increases the fraction of homologous recombinant transformants, albeit at the expense of transformation frequency [116].

The 15.4-Mbp genome of *S. stipitis* was sequenced [73, 155]. *S. stipitis* CBS 6054 is known to have eight chromosomes, of which two pairs are very similar in size, accounting for the earlier results that suggested the presence only of six chromosomes [142]. *S. stipitis* genome annotation predicted that 5841 genes are present in this of which a majority of 72%, have a single exon. The average gene density is 56% and the average gene, transcript, and protein lengths of 1.6 kb, 1.5 kb and 493 amino acids, respectively. Expressed sequence tags (ESTs) confirmed the identity of 40% of the predicted genes with 84% showing strong similarity to proteins in other fungi [73, 155]. So far, protein function can be tentatively assigned to about 70% of the genes. Synteny analysis of *S. stipitis* with its nearest completely sequenced yeast genome neighbor, *Debaryomyces hansenii*, shows extensive recombination and shuffling of the chromosomes, which appear to be a common feature. *S. stipitis* and *D. hansenii* share 151 gene families that are not found in the other genomes. The *S. stipitis* gene set is missing 81 gene families (442 proteins) relative to the other yeast genomes in the analysis.

The most frequent domains characterized so far include protein kinases, helicases, transporters (sugar and MFS), and domains involved in transcriptional regulation (fungal-specific transcription factors, RNA recognition motifs, and WD40 domains). A majority of these are shared with other hemiascomycota. These range from 1534 domains in common with *Schizosaccharomyces pombe* and 1639 domains with *D. hansenii*. One of the few *S. stipitis*-specific domains belong to one of the glycosyl hydrolase families, a subgroup of cellulases and xylanases. All of the genes for xylose assimilation, including the oxidative PPP, glycolytic cycle, the tricarboxylic acid cycle (TCA), and ethanol production, were present in isoforms similar to those found in other yeasts [73, 155]. Genes of the first three enzymes of xylose metabolism, *XYL1, XYL2,* and *XYL3* and that of PPP (*ZWF1, GND1, TKL1, TAL1,* and *RPI1*), are also induced by xylose and the expression of *XYL2* yields one of the most abundant transcripts in xylose-grown cells [74]. Another interesting pattern of the regulation of gene expression was found through oxygen limitation. Such limitation led to strong derepression of some glycolytic genes that include two genes of glyceraldehyde-3-phosphate dehydrogenase *TDH1* and *TDH2*, pyruvate decarboxylase *PDC1* and *PDC2,* and of the secondary alcohol dehydrogenase *SAD2* (the function of the last gene/enzyme in metabolism is not known) [74].

*S. stipitis* has genes for sensing and regulatory proteins (< 200 putative Zn-finger regulatory proteins) which in many cases code for proteins similar to those in *S. cerevisiae.*

The genome *S. stipitis* also revealed many gene clusters representing either pairs/clusters of non-homologous genes in which each cluster has a single function such as galactose metabolism, or tandem repeats of paralogous genes. Gene clusters seem to be particularly abundant in *S. stipitis,* as there are at least 35 clusters of functionally related genes [155]. The studies of structure, function, and regulation in *S. stipitis* genome has an important impact on understanding its physiology and could be used for metabolic engineering of this organism.

The study of *S. stipitis* has attracted scientists and technologists primarily due to its natural ability to produce large amounts of ethanol during xylose fermentation with small or no production of xylitol. It was found that fed-batch cultures of *S. stipitis* produce around 47 g/L of ethanol with yields of 0.36 g/g xylose at 30 °C [182]. However, *S. stipitis* fermentation rate on xylose is low relative to that of *S. cerevisiae* on glucose, so increasing the rate of fermentation of xylose by *S. stipitis* could greatly improve its usefulness in commercial applications [74]. Another drawback to using *S. stipitis* is that it is much more susceptible to ethanol inhibition relative to *S. cerevisiae* [167].

There are many detailed publications on the physiology of *S. stipitis*. Oxygen plays an important role in cell growth, redox balance, functioning of the mitochondria and generation of energy for xylose transport in *S. stipitis* [169]. Fermentation in *S. stipitis* is activated by oxygen limitation [92, 141, 144]. It is interesting to note that *S. stipitis* can metabolize xylose anaerobically, even though it does not grow under anaerobic conditions [197]. The optimal temperature for *S. stipitis* fermentation is between 25 and 33 °C and the optimal pH is in the range of 4.5–5.5 [36]. The nutrients in the fermentation media play an important part in the growth and ethanol production in *S. stipitis*. Ethanol production increased with the addition of amino acids and nitrogen was required for non-growth associated ethanol production [170]. Ammonium salts increased the ethanol productivity and the ethanol to biomass yield in *S. stipitis* [2, 52]. Magnesium has also been shown to play an important role in redox balance and, therefore, has an effect on xylitol production [119]. Low levels of Mg^2+^ resulted in xylitol accumulation and a high intracellular NADH content. Corn steep liquor is a viable nutrient source for *S. stipitis* fermentation when used as a sole nitrogen source compared to amino acids, vitamins, and other nutrients [5]. The initial xylose concentration has an effect on the fermentation parameters of *S. stipitis* with maximum ethanol productivities occurring at a xylose concentration of 50 g/L [37].

The conversion of xylose to ethanol in *S. stipitis* consists of three stages: (i) xylose transport and the initial reaction to enter the PPP; (ii) non-oxidative reactions of PPP; and (iii) glycolysis (Fig. 1). Little is known about xylose transport in *S. stipitis*. The low-affinity transport system is shared between glucose and xylose for sugar transport. Glucose inhibits xylose transport by noncompetitive inhibition in the low-affinity proton symport system [86]. The low-affinity transport is used when sugar concentrations are high and the high-affinity systems are used when sugar concentrations are low. Repression of xylose uptake occurs in fermentation media containing glucose and xylose. Therefore, glucose is the preferred sugar by *S. stipitis* in ethanol production. The rate of glucose consumption is higher than xylose under similar growth conditions [1]. The transport of sugars into the cells is the rate-limiting step in the utilization of sugars for ethanol production in *S. stipitis* [111]. A high-affinity xylose-transporting system has been described that is specific for xylose in this yeast [57]. Three genes*, SUT1, SUT2,* and *SUT3*, have been characterized that encode glucose transporters in *S. stipitis* [195]. Sut2 and Sut3 are highly similar to the *S. cerevisiae* glucose transporter family and the Sut2 and Sut3 transporters have a higher affinity for glucose than for xylose. Transcription of *SUT1* is induced in *S. stipitis* independently of oxygen supply, whereas *SUT2* and *SUT3* are expressed only under aerobic conditions, but independently of the carbon source. Disruption of *SUT1* eliminates the low-affinity xylose transport system in *S. stipitis* [195].

Initial metabolism of xylose in *S. stipitis* is similar to other natural xylose-fermenting yeasts (Fig. 1). Xylose first is reduced by xylose reductase (aldose reductase, gene *XYL1*) to xylitol [183]. This enzyme has affinity to both NADH and NADPH, but shows much higher affinity towards NADPH. By comparison, *S. stipitis* xylitol dehydrogenase (gene *XYL2*), which converts xylitol to xylulose, has affinity only for NAD [122]. The third reaction of xylose metabolism is catalyzed by xylulokinase (gene *XYL3*), which converts xylulose to the PPP intermediate xylulose-5-phosphate [78]. The cofactor imbalance, resulting from the first two reactions involving xylose reductase and xylitol dehydrogenase, leads to xylitol accumulation in most natural xylose-fermenting yeasts, but not in *S. stipitis* [169]. In *S*. *stipitis*, there are efficient systems for NADH reoxidation to NAD and NADPH regeneration [12]. One particular pathway that has been observed for xylose metabolism that is induced under oxygen-limited conditions is effective in tackling the cofactor imbalance caused by the first two steps in xylose utilization [73, 74]. This pathway involves the four enzymes; NAD-dependent glutamate dehydrogenase (*GDH2*), which converts 2-oxoglutarate to l-glutamate consuming NADH; glutamate decarboxylase (*GAD2*), which decarboxylates l-glutamate to 4-aminobutyrate; 4-aminobutyrate aminotransferase (*UGA1*), which transaminates 4-aminobutyrate to succinate semialdehyde; and succinate semialdehyde dehydrogenase (*UGA2*), which oxidizes succinate semialdehyde to succinate using NADP. The net result of the four reactions is the conversion of NADH to NADPH.

The PPP plays an important role in xylose fermentation (Fig. 1) and the corresponding genes (*ZWF1, GND1, TKL1, TAL1, RPI1*) are known to be induced by xylose [74]. Alcohol dehydrogenase is also an important enzyme in ethanol production with the deletion of either *ADH1* or *ADH2* gene significantly reducing ethanol formation, and the deletion of both entirely abolishes ethanol production [22, 143]. Relatively little is known about the rate-limiting steps in ethanol production from xylose. It has been shown that XR and XDH are repressed by glucose and are induced during growth on xylose. Xylose is generally not consumed in the presence of glucose; hence, under glucose repression, these activities, along with xylose transport, are rate limiting [19]. The XK does not, however, appear to be rate limiting in *S. stipitis* once its activity is induced by xylose [78].

In addition to the ultimate goal of using *S. stipitis* for ethanol production from lignocellulosic feedstock, this yeast can also be successfully used for xylitol production from xylose using a mutant defective in *XYL2* that codes for xylitol dehydrogenase [87, 157]. Efficient producers of lactic acid have also been constructed on *S. stipitis* after expression of the lactate dehydrogenase *LDH* gene from *Lactobacillus helveticus* under control of the yeast *ADH1* promoter. It is interesting to note that xylose was more efficient substrate for lactate synthesis than glucose [67]. A strain of *S. stipitis* hyperaccumulating S-adenosylmethionine has been isolated [95]. Other *S. stipitis* recombinant strains have been constructed that produce fumaric acid from xylose after acquiring a heterologous reductive pathway from *Rhizopus oryzae* [194].

In spite of the fact that *S. stipitis* is one of the best natural xylosefermenting yeasts with no xylitol accumulation, it have several drawbacks which include a low rate of fermentation, low tolerance to ethanol and requirement of oxygen for growth. When grown on lignocellulosic hydrolysates, other limitations to the use of this yeast are observed. Among these, there is lack of simultaneous fermentation of glucose and xylose and the poor tolerance to inhibitors that are present in hydrolysates. In addition, as oxygen is required, and *S. stipitis* has a tendency to utilize the ethanol produced when there is still a considerable amount of xylose remaining in the medium [58]. Comparison of the advanced recombinant *S. cerevisiae* strain with the wild-type *S. stipitis* strain regarding xylose fermentation showed that *S. cerevisiae* showed a higher maximum ethanol titer and xylose consumption rate when cultured with a high concentration of xylose, mixed sugars, and under anaerobic conditions than *S. stipitis*. However, its ethanol productivity was less on 40 g/L xylose as the sole carbon source, mainly due to the formation of xylitol and glycerol [168].

One of the most popular methods used to increase *S. stipitis* tolerance towards inhibitors is adaptation by repeated sub-culturing or recycled of yeast cells while increasing concentrations of the inhibitor(s) in a stepwise fashion by adding more concentrated lignocellulosic hydrolysate solutions [4, 65, 193, 202]. Strains adapted to inhibitors present in a specific hydrolysate may exhibit cross tolerance to other hydrolysates. Adaptation has been done to individual inhibitor(s) as well as to mixtures of inhibitors, with the latter approach being most common. Ability to tolerate inhibitory compounds in lignocellulose hydrolysates could reduce the need for detoxification procedures, and this can decrease the overall production cost of ethanol. Adaptation is frequently substituted by random mutagenesis [11] or using genome shuffling via protoplast fusion of strains with different genotypes [9, 10]. To improve ethanol tolerance and production, similar approaches of random mutagenesis and genome shuffling were combined and used [58]. Evolution engineering has been used for selection of *S. stipitis* strains adapted to undetoxified hardwood spend liquor [145]. Selection of higher ethanol tolerance *S. stipitis* strains has been accomplished using UV mutagenesis has also led to improved ethanol production [193]. Protoplast fusion of *S. stipitis* with *S. cerevisiae* has allowed the isolation of a hybrid strain with higher ethanol productivity from xylose relative to the *S. stipitis* parental strain; however, such hybrid was unstable [204]. A popular method for genetic improvement that consists of genome shuffling uses transformation of a yeast strain with total DNA isolated from another strain of the same species or even from different yeast species. In one series of experiments, total DNA of *S. stipitis* was introduced in *S. cerevisiae* by electroporation and the best xylose-fermenting strain isolated was used as the source of DNA for the next round of transformation. This strain showed improvement in ethanol production from xylose and higher ethanol tolerance [209]. To isolate *S. stipitis* strains with improved growth and fermentation characteristics on the xylose/glucose mixture, several fast growing mutants were isolated on a xylose medium with respiration inhibitors antimicyn A and salicyl hydroxamate. Several other mutants have also been isolated which produced more ethanol on xylose/glucose mixture [172]. In another approach, mutants of *S. stipitis* which grow anaerobically on xylose plates were isolated. In contrast to the wild-type strain, these isolated mutants grew and fermented xylose and glucose anaerobically though very slowly [66].

Glucose prevents xylose utilization, as it competes with xylose for transport and its use is subject to glucose catabolite repression. To obtain strains of *S. stipitis* that can ferment simultaneously glucose and xylose, 2-deoxyglucose-resistant mutants were isolated [173]. This was also accomplished by deletion of the gene *HXK1* coding for hexokinase I [26]. The last experiment could be considered as one of very few, in which a metabolic engineering approach was applied for this organism [26]. As a rule, experiments in metabolic engineering of *S. stipitis* are hampered by our limited knowledge of the limiting steps of the fermentation process. In another approach, a mutant of *S. stipitis* with disruption of cytochrome *c* was isolated. Due to defects in respiration, this mutant appeared to be superior when compared to the wild-type strain in xylose alcoholic fermentation, as it accumulated elevated amounts of ethanol [166]. No strains with glucose-insensitive xylose transport have been reported for *S. stipitis*.

To determine the gene(s) affecting *S. stipitis* fermentative capabilities, insertional mutants with altered ethanol production from glucose and xylose have been isolated (M. Semkiv, K. Berezka, K. Dmytruk, V. Passoth, A. Sibirny, unpublished). Mutants obtained by random insertional mutagenesis were screened for their growth abilities on solid media with different sugars and for resistance to the glycolysis inhibitor, 3-bromopyruvate. Fermentations in shake flask agitated cultures were carried out to measure sugar consumption and ethanol formation rates. Subsequently, the most interesting strains were analyzed to determine the genetic background of the observed alterations. Of more than 1300 screened mutants, 17 were identified that have significantly changed ethanol yields during the fermentation. In one of the best fermenting strains, a single insertion event resulted in the enhancement of ethanol formation in the media with both glucose and xylose. This strain had to have within the ORF a gene homologous to *S. cerevisiae* gene YDL119C that encoded for a not yet described mitochondrial transporter and was designated *TMI1* (*T*ransport to *MI*tochondria). Mutant exhibited defects in glucose and xylose respiration. Wild-type phenotype was restored via complementation of the insertion mutation of the wild allele of *TMI1* gene. In addition, the mentioned gene has been deleted and the fragment of *TMI1* gene which is expressed in insertion mutant has been introduced into the deletion *tmi1Δ* strain. It could be suggested that the gene *TMI1* is apparently involved in the control of hexose and pentose alcoholic fermentation in *S. stipitis*.

In summary, *S. stipitis* remains as one of the most efficient organisms for xylose and, in general, lignocellulose fermentation. Access to genome sequence and the development of methods of molecular genetics have been used to alleviate some of the shortcomings of this yeast (low tolerance to ethanol, glucose inhibition of xylose metabolism, and inability to grow anaerobically). This can be accomplished using the combination of rational design methods of metabolic engineering and random selection. Once the before mentioned shortcomings have been addressed, this organism yeast has the potential for use in industrial fermentation.

### *Ogataea polymorpha*

*O. polymorpha* (earlier was designated as *Hansenula polymorph* or *Pichia angusta*) is one of the most thermotolerant yeasts known. It has the ability to grow up to 50 °C. Wild-type strains of *O. polymorpha* are able to ferment glucose, xylose, mannose, maltose, and cellobiose into ethanol, but are not able to utilize and ferment galactose and l-arabinose [160]. Sugar fermentation in this yeast is most efficient under conditions of limited aeration and even at 45–48 °C. At the industrial scale, high-temperature tolerance can reduce cooling costs and the risk of contamination with more energy-efficient removal of ethanol due to lower difference between fermentation and distillation temperatures. Several successful attempts have been made to improve thermotolerance of this yeast even further [70]. It is known that, similar to other fungi, *O. polymorpha* accumulates trehalose and expresses heat shock proteins (Hsps) under heat shock conditions [53]. The increase in the intracellular level of trehalose in *O. polymorpha* following the knock out of acid trehalase gene *ATH1* resulted in a sixfold higher ethanol production of xylose fermentation at 50 °C. The overexpression of the heat shock proteins Hsp16 and Hsp104 has also led to three to six times improved ethanol production at 50 °C [70].

High ethanol tolerance is another important feature of ethanol producers for industrial applications. *O. polymorpha* appears to be more resistant to ethanol than *S. stipitis*; but it is more sensitive than *S. cerevisiae* [32]. Overexpression of the endogenous *ETT1* gene (a homolog of *S. cerevisiae MPE1* gene) significantly increased the resistance of *O. polymorpha* to ethanol, resulting in 10- and 3-fold improvements in the growth on agar and in liquid media with ethanol, respectively. The resistance of *O. polymorpha* to ethyl alcohol was also enhanced by heterologous overexpression of *S. cerevisiae MPR1,* which codes for acetyltransferase [69].

Direct microbial conversion of polysaccharides into ethanol is a promising technology for the production of alcohols from lignocellulosic raw material. The optimal temperature for the activity of hydrolytic enzymes used during microbial conversion of polymers into ethanol is about 50 °C. Recombinant *O. polymorpha* strains that ferment starch were constructed by the expression of heterologous secretory α-amylase and glucoamylase coding for the *SWA2* and *GAM1* genes of *Schwanniomyces occidentalis* [190]. Heterologous expression of *XYN2* of *Trichoderma reesei* and *xlnD* of *Aspergillus niger* that code for secretory endoxylanase and secretory β-xylosidase resulted in strains of *O. polymorpha* with an ability for direct xylan fermentation at high-temperature [185].

*O. polymorpha* has the potential to be used for efficient simultaneous saccharification and fermentation (SSF) due to its temperature tolerance and ability to ferment xylose to ethanol [151]. However, in spite of the robust growth on xylose, the ethanol yield and productivity from this sugar in wild-type strains of *O. polymorpha* are very low (0.03 g/g and 0.02 g/L/h, respectively). Identification of rate-limiting enzymes for xylose conversion to ethanol is necessary for rational strain modification to improve the fermenting efficiency.

The molecular tools for this yeast species are well-developed [45] and a complete genome sequence of the strain NCYC495 of *O. polymorpha* is publically available (http://genome.jgi-psf.org/Hanpo2/Hanpo2.home.html). The genome of *O. polymorpha* has been annotated [155]. As a consequence, a combination of metabolic engineering and classical selection approaches was successfully used to improve the efficiency of xylose alcoholic fermentation in *O. polymorpha* [100].

The difference in cofactor specificity at the first steps of xylose metabolism results in cofactor imbalance causing a substantially reduced ethanol production with accumulation of xylitol as a by-product in yeasts, including that of *O. polymorpha*. The problem arises during consecutive action of NADPH-dependent XR and NAD-dependent XDH that catalyze the reduction of xylose to xylitol and the oxidation of xylitol to xylulose (Fig. 1). To avoid the cofactor imbalance, XR and XDH were replaced with bacterial XI, which directly converts xylose into xylulose with no cofactors required. The bacterial genes *xylA* from *E. coli* or *Streptomyces coelicolor* coding for XI was successfully expressed in a xylose negative *O. polymorpha* strain in which the *XYL1* gene coding for XR and two paralogs *XYL2A* and *XYL2B* of XDH had been deleted. The recombinant strains were able to grow on xylose as carbon source; however, the amount of accumulated ethanol remained at the level of the parental wild-type strain CBS4732 (0.15 g/L) [186]. The overexpression of *E. coli xylA* together with *O. polymorpha XYL3* that codes for XK has led to fourfold increase of ethanol production, but still, the maximal ethanol accumulation did not exceed 0.6 g/L at 48 °C **[**34].

The XR of *O. polymorpha* can use both NADPH and NADH as cofactors. However, the affinity of XR to NADH is significantly lower than that to NADPH. Therefore, another approach to eliminate the imbalance of these two cofactors was based on engineering of the *O. polymorpha* XR with reduced affinity towards NADPH. Using site-specific mutagenesis, a modified XR was constructed by the substitution of lysine and asparagine for arginine and aspartic acid at amino acid positions 341 and 343 [34, 147]. As a result of the modification of the primary structure of the protein, the affinity of XR to NADPH decreased 17-fold as compared to the native enzyme, while the affinity of modified XR to NADH remained unchanged. A recombinant strain of *O. polymorpha* with enhanced expression of modified XR (engineered gene was designated as *XYL1* *m*) and a native XDH and XK was characterized by a fivefold decrease of xylitol accumulation as compared to the wild-type strain and twofold higher ethanol production reaching 1.3 g of ethanol/L [33].

During high-temperature xylose alcoholic fermentation, the wild-type *O. polymorpha* strain NCYC495 can accumulate up to 0.5 g of ethanol/L as compared to the 0.15 g/L seen with strain CBS4732. Pyruvate decarboxylase (PDC) is one of the key enzymes of the final steps of alcoholic fermentation, catalyzing the conversion of pyruvate into acetaldehyde and CO_2_. Subsequently, acetaldehyde is reduced to ethanol by alcohol dehydrogenase (ADH) that is encoded by the *ADH1* gene (Fig. 1). Under conditions of limited aeration, a sufficient activity of PDC is important to redirect pyruvate towards ethanol formation instead of respiration. Overexpression of endogeneous *PDC1* under control of the strong constitutive *GAPp* from glyceraldehyde-3-phosphate dehydrogenase using a plasmid with multicopy integration doubled ethanol production from xylose in the wild-type strain NCYC495 [71]. Overexpression of both *PDC1* and *ADH1* genes in *O. polymorpha* has resulted in an additional twofold activation of xylose alcoholic fermentation when compared to the strain expressing solely *PDC1* [102].

The *O. polymorpha* mutant 2EthOH^−^ unable to utilize ethanol as a sole carbon source was isolated from strain NCYC495 by UV mutagenesis and characterized by a threefold increase in ethanol production from xylose. Subsequently, overexpression of the gene *PDC1* in this mutant^−^ further improved ethanol accumulation, reaching 2.5 g of ethanol/L at 48 ^°^C [71]. To achieve higher ethanol production from xylose, several successful metabolic engineering approaches were combined to modify the genome of the 2EthOH^−^ mutant. The overexpression of the genes *XYL1* *m, XYL2,* and *XYL3*, that code for the modified XR and native XDH and XK, on the background of non-identified mutation in the strain 2EthOH^−^ led to a substantial increase in ethanol accumulation during xylose fermentation (7.4 g/L at 45 °C relative to 0.6 g/L in the wild-type strain NCYC495) [100]. The additional activation of PDC did not lead to any further improvement of xylose conversion to ethanol though the overexpression of *PDC1* on the background of *XYL1* *m* and *XYL2* overexpressed strain, increased ethanol production. The impact of XK on ethanol production during xylose alcoholic fermentation is more pronounced relative to PDC, suggesting that PDC does not limit xylose conversion in strain with higher activities of XR, XDH, and XK.

Mutants selected on a medium supplemented with toxic concentrations of 3-bromopyruvate (BrPA) were characterized by additional increase in ethanol production from xylose (to 10 g/L at 45 ^°^C) [30, 100]. BrPA is known as a compound causing ATP depletion by the inhibition of the glycolytic enzymes, hexokinase II, glyceraldehyde-3-phosphate dehydrogenase and 3-phosphoglycerate kinase [44]. While mutation(s) causing resistance to BrPA in the ethanol overproducing strain remain to be identified, a corresponding mutation was mapped in the strain with the wild-type background. Insertional mutagenesis was used for NCYC495 strain of *O. polymorpha* with subsequent selection of transformants using a mineral medium supplemented with 25 mM of BrPA. Sequencing of the flanking regions revealed that the insertional cassette disrupted the ORF of a gene homologous to the *S. cerevisiae* autophagy-related gene *ATG13* [31]. This gene encodes a regulatory subunit of the Atg1-signaling complex, stimulating Atg1 kinase activity, which is required for vesicle formation during autophagy and the cytoplasm-to-vacuole targeting pathway. The *ATG13* gene mutation led to a 40% increase in ethanol production from xylose as compared to the parental strain. However, the mechanism of such regulation remains unknown [99].

Further possible increase in ethanol yield and productivity from xylose in *O. polymorpha* is hampered by the lack of the knowledge of the regulation of xylose metabolism and fermentation. Therefore, it is important to identify the corresponding genes and, depending on their functions, activate or repress them. Xylose is a unique carbon source that can be fermented to ethanol, similar to glucose, and simultaneously able to be converted to glucose and other hexoses. It is possible mostly in PPP, though partial contribution of gluconeogenesis in hexose synthesis from xylose cannot be neglected. The *CAT8* gene that codes for a zinc-finger cluster protein regulates at least 30 genes involved in gluconeogenesis, ethanol utilization, glyoxylate cycle, and diauxic shift from fermentation to respiration [59]. The roles of *CAT8* gene in the regulation of cell metabolism are well understood in *S. cerevisiae*. It was shown that the deletion of this gene in *S. cerevisiae* and *P. guilliermondii* activated glucose alcoholic fermentation, though maximally achieved level of ethanol in the latter species was still very low [149, 191]. The role of *CAT8* in regulation of xylose metabolism was poorly understood. Transcriptome analysis of the natural xylose-metabolizing yeast *O. polymorpha* did not find changes in *CAT8* expression between xylose- and glucose-containing media [88].

**Fig. 2:**
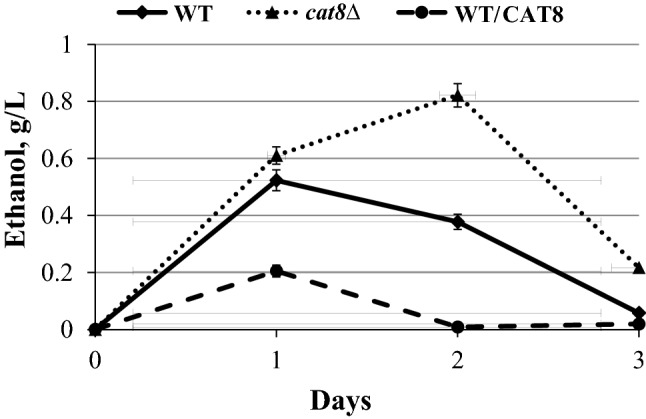
Ethanol production during xylose fermentation of *O. polymorpha* WT, *cat8∆* and WT/CAT8 strains. Alcoholic fermentation of yeast strains was fulfilled by cultivation in liquid mineral medium at oxygen-limited conditions at 45 °C. The conditions were provided by agitation at 140 rpm. 9% xylose was added into the medium used for the fermentation. The cells were pregrown in 100 ml of liquid YPX medium (1% yeast extract, 2% peptone and 4% xylose) in 300 ml Erlenmeyer flasks at 220 rpm till the mid-exponential growth phase. Then the cells were precipitated by centrifugation, washed by water and inoculated into 40 ml of the fermentation medium in 100 mL Erlenmeyer flasks covered with cotton plugs. The initial biomass concentration for fermentation experiments was 2 g (dry weight)/l

To define the role of Cat8 transcriptional factor in xylose fermentation, *O. polymorpha cat8Δ* knock out mutants were constructed and analyzed from either wild type or ethanol overproducing strain (from xylose) [159]. In *O. polymorpha*, *CAT8* deletion did not lead to any significant changes in ethanol production from glucose, while a considerable increase in xylose alcoholic fermentation was observed (Fig. 2). The cell respiration of *cat8Δ* mutants on xylose was impaired to a higher extent relative to that on glucose as a substrate. Moreover, the impaired ethanol and glycerol utilization in *cat8Δ* mutants were observed, suggesting the involvement of *CAT8* in the regulation of gluconeogenesis in *O. polymorpha*, similar to that described for *S. cerevisiae*. Remarkably, growth on xylose of the *cat8Δ* mutant strain was also slightly worsened, suggesting that xylose can be considered, at least partially, as a gluconeogenic substrate. Only slight decrease in the specific activity of fructose-1,6-bisphosphatase in *cat8Δ* mutants was observed, suggesting differences in Cat8 action between *S. cerevisiae* and *O. polymorpha*. It could be suggested that the drop in other enzymes of gluconeogenesis, e.g., phosphoenolpyruvate carboxykinase, explains growth impairment of *O. polymorpha cat8Δ* mutants on gluconeogenic substrates. Overexpression of *CAT8* had the opposite effect on xylose alcoholic fermentation as compared to that in *cat8Δ* mutants, as transformants overexpressing *CAT8* gene were characterized by a decrease in ethanol production from xylose (Fig. 2) [159].

Thus, the *CAT8* gene is one of the first identified genes that are involved in the regulation of xylose alcoholic fermentation in natural xylose-fermenting yeasts. Mutant *O. polymorpha cat8Δ* isolated from the advanced ethanol producer accumulated 30% more ethanol relative to the parental strain, reaching 12.5 g ethanol/L at 45 ^°^C, which is the highest ethanol titer for high-temperature xylose fermentation [159]. The ethanol yield of the constructed *O. polymorpha* recombinant strain (0.34 g/g xylose) is close to that described for *S. stipitis* (0.35–0.44 g/g xylose) [73] and *S. passalidarum* (0.42 g/g xylose) [114]. However, this was achieved for *O. polymorpha* at 45 °C, whereas the compared organisms are mesophilic and, therefore, unable to grow and ferment at such a high temperature. Among the thermotolerant ethanol-producing strains, the most promising one is an engineered *K. marxianus* strain with ethanol yield 0.38 g/g xylose at 42 °C, but lower yield at 45 °C (0.27 g/g xylose). The additional advantage for *O. polymorpha* recombinant strain in contrast to recombinant *K. marxianus* was that no xylitol accumulation can be observed [207].

**Fig. 3:**
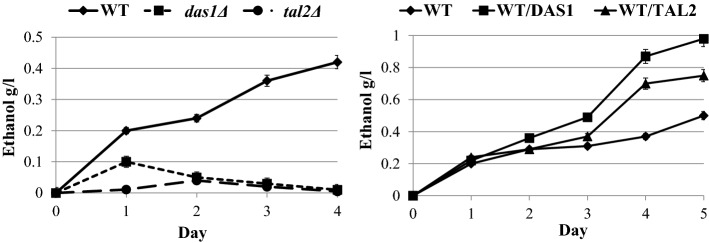
Ethanol production during fermentation of *O. polymorpha* WT, *das1Δ, tal2Δ,* WT/DAS1 and WT/TAL2 strains on xylose containing medium. The fermentation conditions were the same as described in Fig. 2

*O. polymorpha* belongs to the methylotrophic yeasts that are capable for growth on methanol as sole source of carbon and energy. To enable methanol utilization, these organisms have evolved highly specialized metabolic pathways that are partly compartmentalized in peroxisomes. The first enzyme of methanol catabolism, alcohol oxidase, catalyzes oxidation of methanol into the two reactive compounds, formaldehyde, and hydrogen peroxide. Alcohol oxidase is localized in peroxisomes together with catalase, which decomposes hydrogen peroxide into water and oxygen. A third peroxisomal enzyme of methanol metabolism is dihydroxyacetone synthase (DHAS). DHAS is a component of the xylulose-5-phosphate cycle and catalyzes the transfer of two-carbon moieties from xylulose-5-phosphate to formaldehyde with the production of glyceraldehyde-3-phosphate (an intermediate of glycolysis) and dihydroxyacetone, which after phosphorylation is converted to a glycolytic intermediate [181]. DHAS can also display classical transketolase activity using aldose phosphates (such as ribose 5-phosphate) as the acceptors for the glycolyl group from the donor substrate xylulose 5-phosphate, therefore, playing role in xylose utilization through PPP [112]. Screening for other enzymes that are putatively involved in xylose utilization in *O. polymorpha* has revealed a peroxisomal transaldolase coded for the gene *TAL2*. Fluorescent labeling proved the peroxisomal localization of Tal2 protein [101]. The functional roles of peroxisome-localized transaldolase and the specific peroxisomal transketolase in xylose utilization and fermentation in *O. polymorpha* remain unclear. To investigate the role of these enzymes in ethanol production during xylose fermentation, the corresponding genes *DAS1* and *TAL2* were overexpressed in *O. polymorpha* NCYC495 strain under control of the strong constitutive promoter of *GAP1* gene (encodes glycerol-3-phosphate dehydrogenase) using a plasmid for multicopy integration pGLG61 [181]. The recombinant strains overexpressing *DAS1* and *TAL2* revealed 4.6- and 1.5-fold increase in the specific activity of the corresponding enzymes. The overexpression of *TAL2* gene resulted in a 1.5-fold increase in ethanol production at the 4th day of xylose fermentation as compared to the wild-type strain (Fig. 3). The effect of the overexpression of *DAS1* gene was more pronounced. A strain overexpressing *DAS1* gene synthesized 2.3-fold higher amount of ethanol than that of the parental strain after 4 days of xylose fermentation (Fig. 3) [98, 101]. Both *das1Δ* and *tal2Δ* mutants did not show any growth retardation on xylose as carbon source, but were impaired in xylose alcoholic fermentation as compared to the wild-type strain (Fig. 3). Overexpression of *DAS1* and *TAL2* genes in *O. polymorpha* in an advanced ethanol producer increased ethanol production by 40% up to 16 g ethanol/L during xylose alcoholic fermentation at 45 °C [101]. As a consequence, it was shown for the first time that peroxisomal enzymes Das1 and Tal2 are involved in the xylose alcoholic fermentation in *O. polymorpha*; however, the functions of peroxisomes during alcoholic fermentation of xylose require further investigation. It could be concluded that peroxisomal transketolase and transaldolase are important for xylose fermentation, but not for utilization of this sugar. *O. polymorpha* mutants with knock out and overexpression of genes *TKL1* and *TAL1* coding for cytosolic transketolase and transaldolase, respectively, also were constructed. The mutants *tkl1Δ* and *tal1Δ* with knock out of these genes did not grow on xylose as sole carbon source, whereas fermented this pentose to ethanol [101]. These data show different roles of cytosolic and peroxisomal transketolases and transaldolases in xylose utilization and alcoholic fermentation. Overexpression of *TKL1* and *TAL1* genes activated xylose fermentation (O. Kurylenko, K. Dmytruk, A. Sibirny, unpublished). It is also worth to mention that xylose but not glucose fermentation in *O. polymorpha* was blocked in the mutants defective in peroxisome biogenesis *pex3Δ* and *pex6Δ,* whereas *pex3Δ* mutants of the non-methylotrophic yeast *S. stipitis* did not differ from the wild-type strain regarding xylose fermentation [101].

Ethanol yield and the productivity of the best *O. polymorpha* strain with overexpressed genes *DAS1* and *TAL2* at 45 °C are like that described for another thermotolerant yeast *K. marxianus* at 42 °C; however, at 45 °C, *K. marxianus* showed a drop in the mentioned parameters. Moreover, the strain of *O. polymorpha* did not accumulate xylitol during xylose fermentation, whereas *K. marxianus* accumulated large amounts of this by-product [207]. Apparently, the main drawback of the constructed *O. polymorpha* strain is incomplete xylose consumption under fermentation condition. The ethanol yield in the best obtained *O. polymorpha* ethanol producer also is not high enough for economic feasibility. Further improvement of the parameters of alcoholic fermentation of xylose in *O. polymorpha* could be obtained by optimization of the transport of this pentose into cells, amplification of the limiting genes of glycolysis and the PPP, as well as that of the genes determining the resistance to toxic and inhibitory compounds derived after pretreatment of lignocellulosic biomass. At present, the resistance of *O. polymorpha* to toxic products (aldehydes, phenols, acetic and formic acids) accumulated in lignocellulose hydrolysates under the conditions of acidic hydrolysis has not been precisely studied.

## Concluding remarks

Commercial strains of *S. cerevisiae* which are characterized by increased ethanol yield from glucose and sucrose (1G ethanol) have been deployed extensively for industrial production. However, apparently, all of these strains have some drawbacks. Strains of *S. cerevisaie* which accumulate more ethanol by cost of biomass typically show lower robustness and cannot compete with wild-type contaminants during non-sterile production process. Strains that accumulate more ethanol due to lower glycerol production display worse performance being more susceptible to osmotic stress relative to the wild-type strains. Mentioned shortcomings should be overcome by additional metabolic changes.

*S. cerevisiae* strains constructed for the production of 2G ethanol efficiently ferment, in addition to glucose, abundant pentose sugars of lignocellulosic hydrolyzates, xylose, and l-arabinose. Strains fermenting galactose have also been constructed. The strains are also known which could ferment different sugars of hydrolyzates simultaneously due to elaboration of specific xylose transporters and expression of genes responsible for xylose catabolism under control of strong constitutive promoters. Strains resistant to inhibitors of lignocellulosic hydrolyzates are also described. Pilot-plant production of 2G ethanol using engineered *S. cerevisiae* strains has been started.

*S. stipitis* strains with further improvements of xylose fermentation on lignocellulosic hydrolyzates have been constructed. Such strains have defects in glucose catabolite repression and are more resistant to inhibitors present in hydrolyzates. Still, low ethanol tolerance and the need in oxygen for growth are major drawbacks of *S. stipitis* that need to still be addressed. Plans to start pilot plants for 2G ethanol production based on *S. stipitis* are known (T.W. Jeffries, personal communication). If so, such plant could be good platform for further development of *S. stipitis* strains.

*O. polymorpha* also looks as another promising organism for 2G ethanol production especially as it could ferment at temperatures of 45 °C and higher which is compatible with SSF process. However, the level of ethanol production from xylose is still low, glucose inhibits xylose utilization, and fermentation characteristics of glucose and xylose from the real lignocellulosic hydrolyzates are not known. l-arabinose and galactose are not metabolized by *O. polymorpha*. These questions and problems need to be addressed prior to commercial deployment of this yeast.
